# Overcoming constraints to measuring O_2_ diffusivity and consumption of intact roots

**DOI:** 10.1093/plphys/kiae046

**Published:** 2024-02-15

**Authors:** Juan de la Cruz Jiménez, William Armstrong, Timothy D Colmer, Ole Pedersen

**Affiliations:** Department of Biology, University of Copenhagen, Copenhagen 2100, Denmark; Department of Biological Sciences, University of Hull, Hull HU6 7RX, UK; School of Agriculture and Environment, The University of Western Australia, Crawley, WA 6009, Australia; School of Agriculture and Environment, The University of Western Australia, Crawley, WA 6009, Australia; The UWA Institute of Agriculture, The University of Western Australia, Crawley, WA 6009, Australia; Department of Biology, University of Copenhagen, Copenhagen 2100, Denmark; School of Biological Sciences, The University of Western Australia, Crawley, WA 6009, Australia

## Abstract

A method using O_2_ microsensors enables detailed quantification of respiratory O_2_ consumption and diffusive resistance to O_2_ of individual root cell layers.

Dear Editor,

Oxygen is essential for root growth, ion uptake, and cell maintenance and is obtained by radial diffusion from the soil, and/or longitudinally from the shoot system. In shoots, the O_2_ is sourced from photosynthesis or the aerial environment and moves into and along roots through the cortical gas spaces. Supply from the shoot dominates if roots are in an anaerobic environment and O_2_ will then diffuse outward from root to medium viz radial O_2_ loss (ROL) unless prevented by secondary O_2_-impermeable apoplastic barriers and O_2_ consumption in the epidermal-hypodermal layers. Oxygen availability to cells within the root is determined by their position, their O_2_ demands, and those of abutting tissues and by the resistances to diffusion along the supply path. It is sometimes possible to measure the respiratory demands of tissues in vitro using isolated segments of stele, or cortex, or combined peripheral cell layers (e.g. epidermis and hypodermal tissue; Armstrong et al. 1991; Aguilar et al. 2003). However, such data must be used with caution because of wound responses and the disruption to substrate supply and tissue connectivity. Measuring the diffusive resistance to O_2_ through tissues can be even more problematic. Longitudinal gas-phase diffusive resistance through cortical tissue may be readily calculable from gas-filled porosity values, but radial liquid-phase diffusive resistances into the stele or across the peripheral epidermal and hypodermal tissues are not easily measured (Garthwaite et al. 2008; Kotula and Steudle 2009). Here, we propose an innovative approach for measuring both the diffusive resistance and O_2_ consumption of individual peripheral tissue layers in intact roots. Clark-type O_2_ microsensors (Revsbech 1989) will be used to identify the O_2_ concentration deficits across individual cell layers in conjunction with a root sleeving electrode (Armstrong and Wright 1975) that imposes a measurable O_2_ sink for determining the O_2_ flux passing through all the cell layers and not used in respiration (Fig. 1). Both measurements are essential components in the equations for deriving both the respiratory rate (*M*; mol O_2_ cm^−3^ s^−1^) and the apparent O_2_ diffusion coefficient (*D*; cm^2^ s^−1^) across a tissue cylinder (Supplementary File S1). Intact plants will be fitted in a 2-compartment chamber, where the gas composition of the shoot compartment can be regulated. This approach will allow detailed quantification of O_2_ dynamics within root tissues and the specific contribution of the different root cell layers to O_2_ diffusion impedance and respiratory O_2_ consumption.

**Figure 1.**
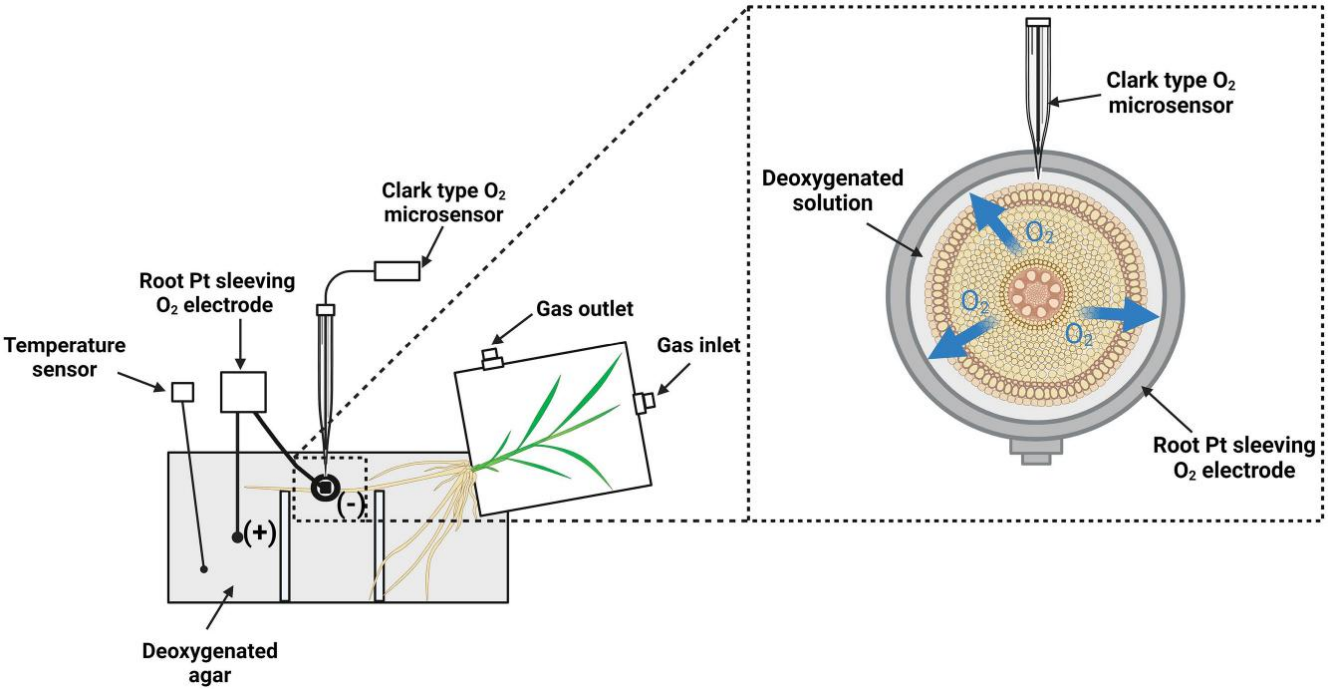
Diagram of the suggested experimental set-up. An intact individual adventitious root is positioned in a wire mesh and along with the remainder of the root system submerged in a deoxygenated agar solution. Shoots of plants are enclosed in a chamber with controlled concentrations of O_2_ (i.e. 21 or 42 kPa). A polarographic platinum (Pt) root-sleeving O_2_ electrode (cathode) is positioned at a specific location along the root and a Clark-type O_2_ microsensor is further inserted through a hole in the Pt electrode and moved in microsteps from the deoxygenated medium down through the different root cell layers. The root-sleeving electrode will generate a fixed O_2_ sink, and a constant readout of total ROL (i.e. outward O_2_ diffusion) from the portion of the root within the electrode, while the microelectrode will measure O_2_ concentrations in the distinct root cell layers. Created with BioRender.com.

Diffusion is the primary mechanism by which gases move within roots (Armstrong 1979). Oxygen can diffuse longitudinally (axially) or radially throughout and across the root (Fig. 2A). Longitudinal diffusion in roots is primarily through the cortical gas spaces (Fig. 2A) where O_2_ diffusivity is high (*D*_O2/air_ = 0.201 cm^2^ s^−1^ at 20 ^°^C). Consequently, longitudinal diffusive resistances, which are readily calculable based on the length (*L*), cross sectional area (*A_x_*) and fractional porosity of the root (ɛ) (viz, *R*_longitudinal_ = *L*/*D*_O2_ ɛ*A_x_*; Armstrong and Armstrong 2014), are relatively low, particularly so in aerenchymatous roots (Fig. 2C). By contrast, much of the radial O_2_ diffusion in roots is within cells and across abutting tissues in the liquid phase where diffusion coefficients are at least 10,000-fold smaller (e.g. *D*_O2/H2O_ = 2.1 × 10^−5^ cm^2^ s^−1^ at 20 °C). Resistances per unit length of path in the liquid phase are therefore much greater than via the cortical gas space and made effectively a further 30-fold greater resistance because of low O_2_ solubility in the liquid phase. In dense tissues (i.e. stele, epidermal, and exodermal layers), O_2_ diffusion can be further reduced by wall deposits which are an additional physical barrier to O_2_ diffusion, and by increased respiratory O_2_ consumption per volume of tissue (Fig. 2C and D). Even without cell wall deposits or respiration, the radial resistance to O_2_ diffusion is calculable as *R*_radial_ = 30 × *r_c_*log_e_*r_r_*/*r_c_*/*D*_O2_*A_c_*: where *r_c_* is the radius of cortex, *r_r_* the root radius, and *A_c_* the surface area on *r_c_* within the segment (Armstrong and Armstrong 2014). For a hypothetical epidermal/hypodermal path of 0.0046 cm in a root segment of 1 cm length and 0.075 cm diameter (Figs. 2C and D) the resistance would be of ca. 2.98 × 10^4^ s cm^−3^, whereas the comparable longitudinal diffusive resistance of a 10 cm, 30% aerenchymatous root of the same diameter would be 3.75 × 10^4^ s cm^−3^. As a consequence, it is not surprising that O_2_ can begin to rise in the stele of such an aerenchymatous root within 2 min of exposing the shoot to double the atmospheric O_2_ concentration (Fig. 2B). Such contrasts in O_2_ consumption and diffusivity strongly influence tissue O_2_ supply across and along roots and are reflected in concentration gradients which can be accessed by O_2_ microsensors (Fig. 2C and D).

**Figure 2.**
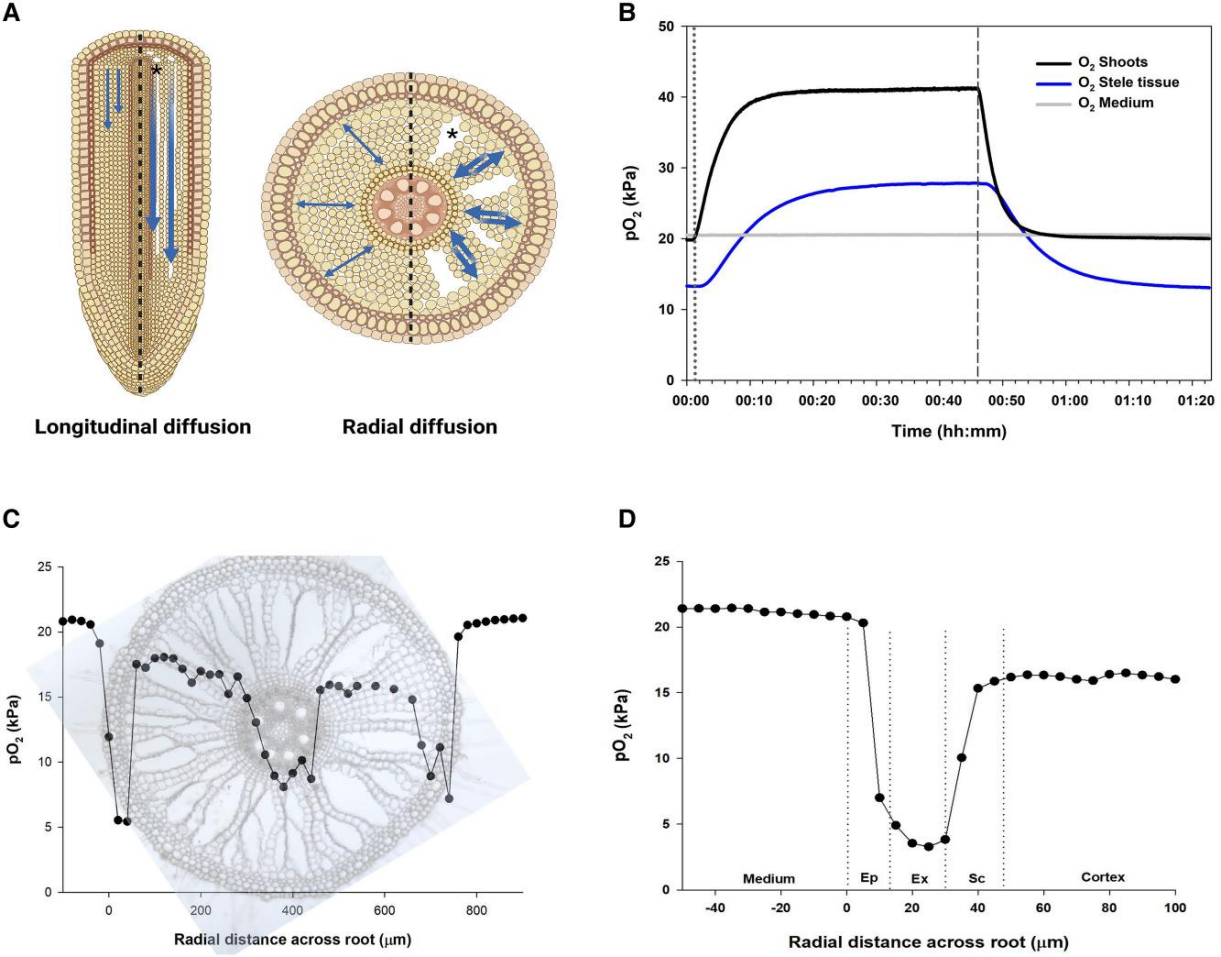
Oxygen dynamics within roots. Longitudinal and radial diffusion of O_2_**(A)**, differences in pO_2_ in the stele tissue when changing the pO_2_ in the shoots **(B)**, pO_2_ profile across adventitious root of intact plants of *Urochloa humidicola***(C)**, and detailed pO_2_ profile taken at the periphery of the root **(D)**. Root characteristics including the number of cell files, stele area, gas-filled spaces, the chemical composition of cells, and the individual respiratory O_2_ consumption determine radial and longitudinal O_2_ diffusion and bulk tissue respiration. Asterisk keys in (A) indicate gas-filled spaces in roots. In (B), the shoot of entire plants of *U. humidicola* was sealed off in a chamber and the roots were placed in a glass chamber filled with stirred water at O_2_ equilibrium (21 kPa). A Clark-type O_2_ microelectrode was inserted in the stele tissue and the pO_2_ in the shoot was changed from 21 to 42 kPa (dotted line) and then changed back to 21 kPa (short dash line). pO_2_ profiles were taken in step sizes of 25 *µ*m or 5 *µ*m in (C) and (D), respectively, starting outside root surface (medium) and ending inside the root tissues. In (D), there is a clear efflux of O_2_ from the cortex outward into the sclerenchyma but the steepness of the gradient is indicative of a very substantial diffusive resistance as does the steepness of the gradient from the medium into the exodermal/epidermal cells. If the medium around the root had been anaerobic, the combination of high diffusive resistance and respiratory demand would probably have presented an almost complete barrier to ROL to the medium. Vertical dotted lines in (D) indicate approximate boundaries of tissue layers. Ep = epidermis, Ex = exodermis, Sc = sclerenchyma.

Current developments in microsensing technologies (Pedersen et al. 2020) allow quantification of O_2_ across root cell layers at sufficient resolution to identify changes in pO_2_ at a nanomolar scale and at individual cell resolution (Fig. 2D). In addition, the development of genetically encoded O_2_ biosensors responding to cellular changes in O_2_ status, ATP levels, NAD redox dynamics or oxidative stress during plant acclimation to different concentrations of O_2_ (Panicucci et al. 2020; Dalle Carbonare et al. 2023) could provide higher resolution of pO_2_ to organelle level. However, the main limitation remains in the translation of such O_2_ profiles/signals into diffusive resistances and respiratory O_2_ consumption by individual cell layers. Any attempt to measure respiration should consider the effects of both O_2_ resistance and consumption. For instance, bulk O_2_ consumption rates of root segments with apoplastic cell wall barriers preventing radial O_2_ diffusion increased 2- to 6-fold when these root sections were sliced open to allow O_2_ diffusion to the entire root cells, in comparison with unopened root sections preventing O_2_ diffusion into the root (Jiménez et al. 2021; Peralta Ogorek et al. 2023). Overlooking the important contribution of barriers to O_2_ diffusion and individual respiratory O_2_ consumption would lead to erroneous estimations of respiratory O_2_ consumption rates, as a merely consequence of O_2_ provision not reaching homogeneously the entire tissues.

Models for root aeration (Armstrong and Beckett 1987; Armstrong et al. 1991) represent the closest approximation to understanding how O_2_ resistances and consumption in roots affect oxygen supply and distribution, but experimentally determined input values for tissue diffusivities and respiratory activities in roots are scarce. The method proposed here (see Supplementary File S1), will allow quantification of pO_2_ across individual cell layers and the throughflow diffusion across these cell layers, which will allow both the respiratory rate and the apparent O_2_ diffusion across each of the different cell layers of a root to be derived.

The acquisition of detailed information on radial differences in O_2_ demand is essential for understanding root aeration, respiration, and nutrient uptake. High-resolution O_2_ consumption of individual root cells would allow a comprehensive characterization of cell growth and energy-driven ion transport, where higher ATP requirements are probably needed in developing cells and at ion entry points located at exodermal and endodermal cells. Moreover, a complete understanding of O_2_ dynamics in roots and the influence of root anatomy on respiratory O_2_ consumption would allow the scientific community to characterize in detail plant responses to anoxic conditions (flooded soils or submergence), O_2_ limitations during postharvest, or the role of O_2_ in developmental processes (cf. Weits et al. 2019).

## Supplementary Material

kiae046_Supplementary_Data

## Data Availability

All data supporting the findings of this study are available in the main paper and supplementary data.

## Supplementary data

**Supplementary File S1.** Mathematical modeling to quantify resistances and respiratory O_2_ consumption of individual root layers.

## Dive Curated Terms

The following phenotypic, genotypic, and functional terms are of significance to the work described in this paper:

oxygen CHEBI: CHEBI:25805

ATP CHEBI: CHEBI:15422

NAD CHEBI: CHEBI:13389

epidermis AmiGo: PO:0005679

root AmiGo: PO:0009005

stele AmiGo: PO:0025197
