# Supplementary material for: Overcoming constraints to measuring O2 diffusivity and consumption of intact roots
Source: Plant Physiol. 2024 Feb 15;195(1):283–6. doi: 10.1093/plphys/kiae046 (PMC11060671; doi:10.1093/plphys/kiae046)
Supplement: kiae046_Supplementary_Data [file kiae046_supplementary_data.pdf]

Supplemental File S1. Mathematical modeling to quantify resistances and respiratory O2 consumption of individual root layers.

The following approach can be used to measure respiratory O_2 consumption and diffusive resistances through root tissues. It can be adopted to multiple roots independently of the specific cell file number or root diameters and it should work at all points along a root provided that there is radial O_2 loss, and that the microelectrode can penetrate the root.

Mathematical modeling

Assuming a root with the following multi-cylindrical radial composition:

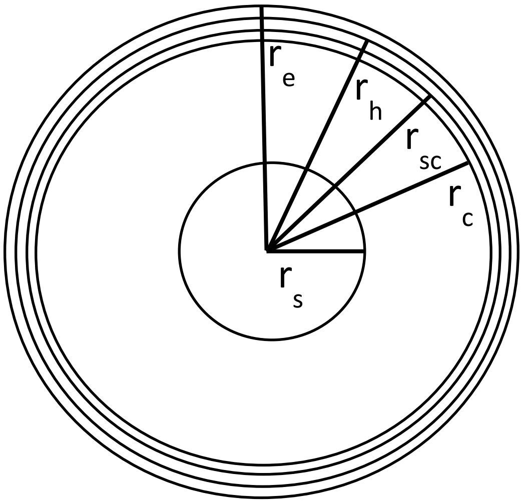

where r_s= stele radius, r_c= cortex radius, r_{sc}= sclerenchyma radius, r_h= hypodermis/exodermis radius and r_{ep}= epidermis radius.

According to the model for ***inward diffusion*** of O_2 to a root from an aerated rooting medium (Armstrong et al., 1991), the total O_2 deficit (ΔT) radially across the root can be expressed by the following equation:

$$\Delta T= \Delta's + (\Delta'c +\Delta''c) + (\Delta'sc + \Delta''sc) + (\Delta'h + \Delta''h) + (\Delta'e + \Delta''e) \quad \ldots\ldots\ldots (1)$$

where Δ' represents an O_2 deficit caused by O_2 consumption *in situ* within the specific tissue cylinder and Δ'' represents a deficit caused by O_2 *'throughflow'* to other cell layers, e.g., Δ''e will be determined by diffusion through the epidermis to the hypodermis, sclerenchyma, cortex and stele, Δ''h by flow through the hypodermis to the sclerenchyma, cortex and stele, and so on.

For roots in waterlogged soils or (experimentally) in stagnant anaerobic agar, where oxygen is sourced from that diffusing down the cortex from the shoot, a similar approach can be applied

to the ***outward O_2 diffusion*** from the cortex and through the outer cell layers (OCL) to the medium. If in addition the root is sheathed at some point by a polarised sleeving polarographic platinum (Pt) electrode (Armstrong and Wright, 1976) its inner surface will maintain an O_2 concentration of zero at radius of Pt sleeving electrode (r_{Pt}), inducing ROL from the root and thus acting as a fixed quantifiable O_2 sink. In this case, the contribution of the OCL and the electrode to the decline in O_2 concentration between the outer perimeter of the cortex and the electrode can be described by the following equation:

$\Delta T = C_c - C_{Pt} = (\Delta'sc + \Delta''sc) + (\Delta'h + \Delta''h) + (\Delta'e + \Delta''e) + \Delta_{LS}$ ………………………………………….. (2)

Where C_c is the *liquid-phase* O_2 concentration on the cortical perimeter (r_c), C_{Pt} is the O_2 concentration at the electrode surface, Δ_{LS} is the ROL-induced deficit across the liquid shell between root and electrode caused by consumption at the electrode surface. The Δ'' concentration drops arise due to O_2 *throughflow* to outer cell layer(s) and the electrode. In case of $\Delta''e$ this drop is due only to O_2 consumption by the electrode.

We can expand on the various deficits (Δ') and (Δ'') in terms of their respiratory activities (M), O_2 diffusivities (D) and radial dimensions, and for the sclerenchyma cell layer it becomes:

$\Delta sc' = [M_{\mathbf{sc}}\, r_{\mathbf{sc}}^2/4D_{sc}\, (r_c^2/r_{sc}^2 + 2\log_e r_{sc}/r_c - 1)]$: the concentration drop (mol cm^{-3}) across the sclerenchyma layer due to respiration, $M_{\mathbf{sc}}$ (mol $cm^{-3}\, s^{-1}$) within the sclerenchyma layer itself and D_{sc} is the effective O_2 diffusion coefficient ($cm^2\, s^{-1}$) (for derivation see Armstrong, 1979) ……... (3)

$\Delta sc'' = [(M_h/2D_{sc})(r_h^2 - r_{sc}^2)\, (\log_e r_{sc}/r_c)]$: the concentration drop in the sclerenchyma layer due to O_2 used by respiration in the hypodermis (M_h) ……………….. (4a)

$+ [(M_e/2D_{sc})\, (r_h^2 - r_{sc}^2)\, (\log_e r_{sc}/r_c)]$: the concentration drop in the sclerenchyma layer due to O_2 used by respiration in the epidermis (M_e)………………………(4b)

$+$ ROL $(r_c \log_e r_{sc}/r_c)/D_{sc}A_c$: the concentration drop across the sclerenchyma due to the O_2 consumed by the electrode which leaves the root as ROL …………..(4c)

Equations 4a,b and c are based on simple diffusion between cylinders according to the Fick's Law equation $\Delta C = J\, R$, where ΔC is the concentration drop between the cylinders, J is the diffuson rate between the cylinders (mol s^{-1}), and R the resistance given as L/DA (s cm^{-3}), where L is the effective diffusive path length (cm) between the cylinders and given by $r_i \log_e r_o/r_i$, and D_{sc}, the effective diffusion coefficient. Since these are cylindrical cell layers within the electrode (length for example h=0.5 cm), then for the effect of the hypodermal cylinder (Eq. 4a): $\Delta C = M_h \times 0.5\pi(r_h^2 - r_{sc}^2)$ mol s^{-1} is multiplied by $(r_c \log r_{sc}/r_c)\, /(2\, D_{sc}\pi\, r_c\, 0.5)$ s cm^{-3} and this simplifies to $[(M_h/2D_{sc})(r_h^2 - r_{sc}^2)(\log_e r_{sc}/r_c)]$ mol cm^{-3}. The throughflow deficit due to the epidermis (Eq. 4b) is derived

similarly. For equation 4c the radial O_2 loss (ROL) will be 4.974 × μA / 1920 nmol s^{-1} (Armstrong, 1979).

Similarly, the deficit across the hypodermis will be:

$$C_{sc} - C_h = [M_h r_h^2 / 4D_h (r_{sc}^2/r_h^2 + 2\log_e r_h/r_{sc} - 1)] + [(M_e/2D_{sc})(r_e^2 - r_h^2)(\log_e r_{sc}/r_c)] + ROL (r_{sc} \log_e r_h/r_{sc}) / D_h A_{sc} \quad \ldots\ldots\ldots (5)$$

For the epidermis it is:

$$C_h - C_e = [M_e r_e^2 / 4D_e (r_h^2/r_e^2 + 2\log_e r_e/r_h - 1)] + ROL (r_h \log_e r_e/r_h) / D_e A_h \quad \ldots\ldots\ldots (6)$$

And for the liquid shell (LS) between the root and electrode surface at r_{Pt}:

$$C_e - C_{Pt} = C_e = ROL (r_e \log_e r_{Pt}/r_e) / D_{LS} A_e \quad \ldots\ldots\ldots (7)$$

Where D_{LS} is the O_2 diffusion coefficient of the liquid shell.

Protocol 1 - Description of modeling approach

Determining M and D values for each cell layer is very challenging and to introduce the principles involved we will first treat the sclerenchyma, hypodermal and epidermal layers as one entity – outer cell layers (OCL). To obtain an overall measure of O_2 diffusive resistance across them, it is necessary to eliminate any effect of respiratory O_2 consumption. To this end, a modification of the two-point gas-mixture method of Armstrong and Wright (1975) for estimating root pore-space resistance in intact plants may be used. The method makes no attempt to curtail respiration, but eliminates its influence by measuring ROL using a root-sleeving Pt electrode at each of two shoot-sourced-O_2 concentrations sufficiently high enough to saturate root respiration. It has been shown previously that the critical O_2 pressure for respiration at the cellular level in intact roots can be very low (viz. ≤ 2 kPa: Armstrong et al., 2009; Armstrong and Beckett, 2011). Consequently, shoot O_2 concentrations should be chosen to maintain O_2 levels in the root substantially higher than this at all times. Two simultaneous equations that describe ROL in terms of the cortical O_2 concentration (C_{c2} and C_{c1}), OCL resistance, LS resistance between root and electrode surface, and respiration can then be combined to eliminate the respiratory component. Concentrations C_{c2} and C_{c1} would be determined as the mean of microelectrode measurements made at the cortex-hypodermal boundary (r_c) immediately above and below the sleeving electrode.

The two equations are:

$ROL_1 = [C_{c1} / (R_{OCL} + R_{LS})] – Resp$ .... (8)

$ROL_2 = [C_{c2} / (R_{OCL} + R_{LS})] – Resp$ .... (9)

where C_{c1} = cortical *liquid-phase* O_2 concentration on r_c for treatment 1 (e.g., shoots in air, 21% O_2) and C_{c2} = cortical liquid-phase O_2 concentration on r_c for treatment 2 (e.g., shoots in 42% O_2). ROL_1 and ROL_2 are the radial O_2 loss rates (nmol s^{-1}) corresponding with these treatments, R_{OCL} = resistance to O_2 diffusion across the external cell layers (s cm^{-3}), R_{LS} = resistance to O_2 diffusion in the liquid agar between the root surface and the electrode surface = $[(r_e \log_e r_{Pt}/r_e) / D_{LS} A_e]$, and Resp is respiratory component.

Since the respiration is the same in both equations, then:

$ROL_2 - ROL_1 = C_{c2} - C_{c1} / (R_{OCL} + R_{LS})$,
$C_{c2} - C_{c1} / (ROL_2 - ROL_1) = R_{OCL} + R_{LS}$,
$\therefore R_{OCL} = [C_{c2} - C_{c1} / ROL_2 – ROL_1] - (R_{LS})$ .... (10)

Data for the right-hand side of equation 10 are found as follows:

ROL_1 and ROL_2, the data from the root-sleeving O_2 electrodes, are expressed as diffusion rate (nmol s^{-1}) using the following (Armstrong, 1979):

$ROL = 4.974 (I_{diff} – I_{resid})/1920$ .... (11)

where I_{diff} = equilibrium current (μA) generated by electrolytic O_2 reduction at the electrode surface, and I_{resid} = residual current (close to zero) where the root is replaced by a glass rod of similar dimensions to the root.

R_{LS}, the resistance to O_2 diffusion in the liquid agar between the root surface and the electrode surface (s cm^{-3}), is calculated using the following equation (Armstrong & Wright, 1975):

$R_{LS} = [r_e \ln(r_{Pt}/r_e)/(D_w A_e)]$ .... (12)

where D_w = diffusion coefficient for O_2 in water at measurement temperature and A_e = surface area of the root within the electrode (cm^2).

The ***apparent diffusion coefficient*** for the OC layers, $\boldsymbol{D_{OCL}}$, can then be determined from the following:

$R_{OCL} = r_c \ln (r_e/r_c) / D_{OCL} A_c$ …………………………………………………………………….... (13)

$\mathbf{D_{OCL}} = r_c \ln (r_e/r_c) / R_{OCL} A_c$, ……………………………………….……………………. (13a)

where $A_c = 2\pi r_c 0.5 = \pi r_c$

The ***apparent respiratory rate*** for the OC layers, $\boldsymbol{M_{OCL}}$, can be determined from the following:

$C_c – C_e = [M_{OCL} r_c^2 / 4D_{OCL} (r_c^2/r_e^2 + 2\log_e (r_e/r_c) - 1)] + ROL (r_c \log_e r_e/r_c) / D_{OCL}A_c$ …….. (14)

where C_e is the corresponding root surface O_2 concentration for either C_{C2} or C_{C1} and is derived from the corresponding ROL using the expression $C_e = ROL (r_e \log_e r_{Pt}/r_e) / D_{LS} A_e$

Protocol 2 - Individual resistances and respiratory O_2 consumption of root cells.

This is the protocol designed to measure independently the O_2 diffusivities and respiratory activities of each of the 3 cell layers: the sclerenchyma, the hypodermis/exodermis, and the epidermis. Again, the two-point gas-mixture method is used but the O_2 concentrations on r_C, r_{sc}, r_h and r_e are measured by means of a Clark-type microelectrode inserted through a narrow hole in the side wall of the sleeving electrode. At each of the two shoot O_2 applications, when the ROL has stabilised, the microelectrode is tracked radially into the root from r_e to r_c in µm steps recording the O_2 concentration at each point.

The concentrations on r_c, r_{sc}, r_h and r_e may only be reliably identifiable when the microelectrode data can be compared alongside the subsequent anatomical sections revealing the tracks across the OC layers. On the other hand, if the thicknesses of the OCLs can be reasonably predicted it may be that knowing by how far the electrode has tracked, or by some obvious indication from the readings that the cortex has been reached, then it may be that you can reasonably assign O_2 concentrations to r_C, r_{sc}, r_h and r_e before sectioning.

From Eq. 10 and knowing C_{2c} and C_{1c} from the microelectrode readings, we obtain:

$R_{OCL} = R_{sc} + R_h + R_e = [(C_{2c} – C_{1c}) / (ROL_2 – ROL_1)] - R_{LS}$ ………………………………... (15)

and, knowing the concentrations C_{2h} and C_{1h} from the microelectrode readings then:

$R_e = [(C_{2h} – C_{1h}) / (ROL_2 – ROL_1)] - R_{LS}$ ………………………………………………………... (16)

Similarly, $R_h = [(C_{2sc} – C_{1sc}) / (ROL_2 – ROL_1)] – (R_e + R_{LS})$ ……………………………………….... (17)

and $R_{sc} = R_{OCL} - (R_h + R_e + R_{LS})$ ……………………………………………………………………… (18)

Since we now know the O_2 diffusive resistances, R_{sc}, R_h, and R_e we can determine the effective diffusion coefficients D_{sc}, D_h, and D_e from appropriate variants of equation 13a, viz:

$$\mathbf{D_{sc}} = r_c \ln (r_{sc}/r_c) / R_{sc} A_c \quad \text{……(19)},$$

where $A_c = 2\pi r_c 0.5 = \pi r_c$

$$\mathbf{D_h} = r_{sc} \ln (r_h/r_{sc}) / R_h A_{sc} \quad \text{……(20)},$$

where $A_h = 2\pi r_{sc} 0.5 = \pi r_{sc}$

$$\mathbf{D_e} = r_h \ln (r_e/r_h) / R_e A_h \quad \text{……(21)},$$

where $A_e = 2\pi r_h 0.5 = \pi r_h$.

To determine the apparent respiratory rates for the OCLs we need to refer back to equations 3 to 7. Since we now know D_e, and a C_h and C_e for each of the two shoot-sourced-O_2 concentrations, we can now apply Eq.6 and solve for $\mathbf{M_e}$.

$$C_h - C_e = [\mathbf{M_e}\, r_e^2/ 4D_e (r_h^2/r_e^2 + 2\log_e r_e/r_h - 1)] + ROL\, (r_h \log_e r_e/r_h)/ D_eA_h \quad \text{……(22)}$$

$$\therefore \mathbf{M_e} = [(C_h - C_e) - ROL\, (r_h \log_e r_e/r_h)/ D_eA_h] / [r_e^2/ 4D_e (r_h^2/r_e^2 + 2\log_e r_e/r_h - 1)] \text{……(23)}$$

Having determined M_e, D_e, and D_h and experimentally recorded a C_{sc} and C_h for each of the two shoot-sourced-O_2 concentrations we can now apply Eq. 5 and solve for M_h, viz.:

$$C_{sc} - C_h = [\mathbf{M_h}\, r_h^2/ 4D_h (r_{sc}^2/r_h^2 + 2\log_e r_h/r_{sc} - 1)] + [(M_e/2D_{sc})(r_e^2-r_h^2)(\log_e r_{sc}/r_c)]$$
$$. + ROL\, (r_{sc} \log_e r_h/r_{sc})/ D_hA_{sc} \text{……(24)}$$

$$\therefore \mathbf{M_h} = \{(C_{sc} - C_h) - [(M_e/2D_{sc})(r_e^2-r_h^2)(\log_e r_{sc}/r_c)] - [ROL\, (r_{sc} \log_e r_h/r_{sc})/ D_hA_{sc}]\}$$
$$/ [r_h^2/ 4D_h (r_{sc}^2/r_h^2 + 2\log_e r_h/r_{sc} - 1)] \quad \text{…(25)}$$

Having determined M_e, M_h, D_e, D_h and D_{sc}, and experimentally recorded a C_c and C_{sc} for each of the two shoot-sourced O_2 concentrations we can now combine Eq. 3, 4a, 4b and 4c and solve for M_{sc}, viz.:

$$C_c - C_{sc} = [\mathbf{M_{sc}}\, r_{sc}^2/4D_{sc} (r_c^2/r_{sc}^2 + 2\log_e r_{sc}/r_c - 1)] + [(M_h/2D_{sc})(r_h^2-r_{sc}^2)(\log_e r_{sc}/r_c)]$$
$$+ [(M_e/2D_{sc})(r_h^2-r_{sc}^2)(\log_e r_{sc}/r_c)] + ROL\, (r_c \log_e r_{sc}/r_c)/D_{sc}A_c \text{ ……(26)}$$

$$\therefore \mathbf{M_{sc}} = \{(C_c - C_{sc}) - [(M_h/2D_{sc})(r_h^2 - r_{sc}^2)(\log_e r_{sc}/r_c)] - [(M_e/2D_{sc})(r_h^2 - r_{sc}^2)(\log_e r_{sc}/r_c)] - [ROL\ (r_c \log_e r_{sc}/r_c)/D_{sc}A_c]\} / [r_{sc}^2/4D_{sc}\ (r_c^2/r_{sc}^2 + 2\log_e r_{sc}/r_c - 1)] \quad \text{………………(27)}$$

References

Armstrong W, Wright EJ (1975) Radial Oxygen Loss from Roots: The Theoretical Basis for the Manipulation of Flux Data Obtained by the Cylindrical Platinum Electrode Technique. Physiologia Plantarum **35:** 21-26

Armstrong W, Wright EJ (1976) An electrical analogue to simulate the oxygen relations of roots in anaerobic media. Physiologia Plantarum **36:** 383-387

Armstrong W (1979) Aeration in higher plants. *In* W H.W.W., ed, Advances in Botanical Research, Vol 7. Academic Press, pp 225-332

Armstrong W, Webb T, Darwent M, Beckett PM (2009) Measuring and interpreting respiratory critical oxygen pressures in roots. Ann Bot **103:** 281-293

Armstrong W, Beckett PM (2011) Experimental and modelling data contradict the idea of respiratory down-regulation in plant tissues at an internal [O_2] substantially above the critical oxygen pressure for cytochrome oxidase. New Phytol **190:** 431-441
